# Role of the ROS/AMPK signaling pathway in tetramethylpyrazine-induced apoptosis in gastric cancer cells

**DOI:** 10.3892/ol.2013.1403

**Published:** 2013-06-14

**Authors:** BO YI, DAN LIU, MING HE, QIYUN LI, TIANDE LIU, JIANGHUA SHAO

**Affiliations:** 1 Jiangxi Provincial Key Laboratory of Molecular Medicine, The Second Affiliated Hospital of Nanchang University, Nanchang, Jiangxi 330006;; 2 First Abdominal Surgery Department, Jiangxi Province Tumor Hospital, Nanchang, Jiangxi 330029;; 3 Department of Pharmacology and Molecular Therapeutics, Nanchang University School of Pharmaceutical Science;; 4 Department of General Surgery, The Second Affiliated Hospital of Nanchang University, Nanchang, Jiangxi 330006, P.R. China

**Keywords:** AMP-activated protein kinase, reactive oxygen species, tetramethylpyrazine, apoptosis, mitochondria, gastric cancer cell

## Abstract

Tetramethylpyrazine (TMPZ) is one of the active compounds extracted from the traditional Chinese medicinal herb Chuanxiong and several studies have shown it to possess anticancer properties. However, its effectiveness in gastric cancer and its cellular mechanisms are relatively unknown. The present study aimed to investigate the effect of TMPZ on SGC7901 cells, and it was demonstrated that a high dose of TMPZ inhibited cell viability and induced apoptosis by stimulating AMP-activated protein kinase (AMPK) through the generation of reactive oxygen species (ROS). Furthermore, TMPZ-induced apoptosis resulted in the sequential events beginning with the translocation of Bax, the collapse of mitochondrial membrane potential (ΔΨm), the release of cytochrome *c* and the activation of caspase-9 and -3. Each of these events was inhibited by compound C, a pharmacological inhibitor of AMPK. To the best of our knowledge, these results demonstrate for the first time that the induction of apoptosis by TMPZ in gastric cancer cells is associated with the activation of the ROS/AMPK pathway. AMPK activation induces apoptosis through the mitochondrial apoptotic pathway. In addition, these results raise the possibility that TMPZ may have a future therapeutic role in gastric cancer.

## Introduction

As surgery and adjuvant chemotherapy are associated with low survival rates, there is an urgent requirement to identify novel therapeutic agents for the treatment of gastric cancer. Recently, traditional Chinese medicines have been receiving increasing attention due to their anticancer properties. Tetramethylpyrazine (TMPZ), a major bioactive component of the traditional Chinese medicine Chuanxiong, has been widely used in the clinical treatment of neurovascular and cardiovascular diseases (1,2). Although a number of studies have demonstrated that TMPZ inhibits proliferation and induces apoptosis in rat C6 glioma and human adenocarcinoma cells (3,4), to the best of our knowledge, there are no studies reporting its effects in gastric cancer. The exact mechanism of action of TMPZ is not fully understood.

AMP-activated protein kinase (AMPK) is a metabolic-sensing protein kinase that is important in regulating cellular homeostasis and protecting cells under conditions of metabolic stress (5). AMPK is activated in response to the phosphorylation of the critical amino acid residue Thr^172^(6). Several studies have identified a notable pro-apoptotic potential for AMPK in its activated form (7,8). It has been observed that the activation of AMPK is frequently accompanied by an increase in the levels of reactive oxygen species (ROS); however, the correlation between the levels of ROS and AMPK activation in TMPZ-induced apoptosis is unclear.

In the present study, we examined the effects of TMPZ on the SGC7901 human gastric cancer cell line originating from a human gastric adenocarcinoma. This cell line is widely used in China. Additionally, we examined the key signaling pathways that are stimulated during apoptotic cell death following TMPZ treatment, and investigated whether the inhibition of AMPK activation through pharmacological methods affects these signaling pathways.

## Materials and methods

### Materials

TMPZ was acquired from the National Institute for the Control of Pharmaceutical and Biological Products (Beijing, China). TMPZ was dissolved in distilled water to produce a stock solution of 1 M concentration. RPMI-1640 medium was purchased from Gibco-BRL (Carlsbad, CA, USA). Fetal calf serum (FCS) was obtained from HyClone (Logan, UT, USA). Compound C was purchased from Merck KGaA (Darmstadt, Germany). N-acetylcysteine (NAC), trypsin, 3-(4,5-dimethylthiazol-2-yl)-2,5-diphenyl tetrazolium bromide (MTT), sodium dodecyl sulfate (SDS), Triton X-100, HEPES, NP-40, phenylmethylsulfonyl fluoride (PMSF), aprotinin and leupeptin were obtained from Sigma (St. Louis, MO, USA). The anti-phospho-AMPK (Thr^172^), anti-AMPKα, anti-cytochrome *c* and anti-Bax antibodies were purchased from Cell Signaling Technology, Inc. (Danvers, MA, USA). The anti-caspase-3 and -9 and anti-β-actin antibodies were purchased from Santa Cruz Biotechnology, Inc. (Santa Cruz, CA, USA). All the reagents used in the study were of the highest purity.

### Cell culture

The SGC7901 human gastric adenocarcinoma cell line was obtained from the Chinese Academy of Medical Sciences Cell Center of Basic Medicine (Beijing, China). The cells were maintained in RPMI-1640 medium containing 10% FCS at 37°C, in a humidified incubator with a mixture of 95% air and 5% CO_2_.

### Cell viability assay

Cell viability was analyzed using the MTT assay. Briefly, SGC7901 cells were seeded at 1×10^4^ cells/well in flat-bottomed 96-well plates. At 24 h following TMPZ treatment, 100 *μ*l MTT was added to each well and the cells were incubated at 37°C for 4 h, resulting in the formation of MTT formazan crystals. Following incubation, the supernatants were removed and the formazan crystals were solubilized in dimethylsulfoxide. The plates were thoroughly shaken and the absorbance of each well was measured at 570 nm using a Bio-Tek microplate reader (Bio-Rad, Hercules, CA, USA).

### Analysis of cell apoptosis by flow cytometry

Apoptosis was identified by flow cytometry with an Annexin V-FITC Apoptosis Detection kit (BD Biosciences, San Diego, CA, USA) according to the manufacturer’s instructions. The cells were washed twice with cold phosphate-buffered saline (PBS) and were then resuspended in a buffer solution at a final concentration of 1×10^6^ cells/ml. Annexin V-FITC (5 *μ*l) and PI (5 *μ*l) were added to the cells, which were resuspended in 500 *μ*l of 1X binding buffer. The cells were gently vortexed and incubated in the dark at room temperature for 15 min. The cells were then analyzed using a FACSAria™ flow cytometer (BD Biosciences) within 1 h. A minimum of 10,000 cells was analyzed in each treatment group and the analysis of apoptotic cells was performed using CellQuest software (BD Biosciences).

### Western blot analysis

The cells were rinsed twice with ice-cold PBS and then incubated for 30 min in lysis buffer (50 mM Tris-HCl, pH 8.0; 1% sodium deoxycholate; 1% NP-40; 0.1% SDS; 1 mM PMSF; 10 *μ*g/ml aprotinin and 10 *μ*g/ml leupeptin). The cells were then scraped and centrifuged at 12,000 × g for 10 min at 4°C. Extracts of the cytoplasm and mitochondria were prepared using the Mitochondria/Cytosol Isolation kit (Applygen Technologies, Beijing, China) according to the manufacturer’s instructions. The protein concentration in the lysates was quantified using a BCA Protein Assay kit (Pierce Biotechnology, Inc., Rockford, IL, USA). Cell lysates and fractionated extracts were separated by SDS-polyacrylamide gel electrophoresis and transferred onto nitrocellulose membranes. The membranes were blocked with 5% non-fat dried milk in 10 mM Tris (pH 7.5), 100 mM NaCl and 0.1% Tween-20 for 2 h at room temperature. The proteins were probed with antibodies against the target proteins and the protein bands were visualized by exposure to X-ray film. The bands were quantified by scanning the film on a GDS-8000 UVP photo scanner (Bio-Rad). The monoclonal anti-β-actin antibody was used as an internal control for loading.

### Measurement of intracellular ROS

The levels of ROS were measured using a Reactive Oxygen Species Assay kit (Applygen Technologies). Briefly, the intracellular formation of ROS in the SGC7901 cells was determined using 2′7′-dichlorodihydrofluorescein diacetate (DCFH-DA) molecular probes. DCFH-DA is a membrane-permeable indicator of ROS levels, and is non-fluorescent until the acetate groups are removed by intracellular esterases and oxidation occurs within the cell. Thus, a reaction between these probes and intracellular ROS yields the fluorescent molecule DCF, which may be used as a measure of intracellular ROS levels. Briefly, SGC7901 cells were harvested, washed with PBS and loaded with 10 *μ*M DCFH-DA in serum-free DMEM in the dark for 30 min at 37°C. The excess dye was flushed off and the cells were resuspended in serum-free DMEM. Fluorescence intensity was quantified using a BD FACSVantage™ SE (BD Biosciences).

### Detection of the mitochondrial membrane potential (ΔΨm)

The ΔΨm was evaluated using a Mitochondrial Membrane Potential Assay kit with JC-1 (Beyotime Biotechnology Co., Jiangsu, China). JC-1 is a mitochondria-specific probe and a fluorescent cationic dye that exhibits potential-dependent accumulation in mitochondria by a fluorescence emission shift from green (530 nm) to red (590 nm). Consequently, mitochondrial depolarization is indicated by a decrease in the red-green fluorescence intensity ratio. The cells were plated at a density of 5×10^5^ cells/well in 6-well plates. Following treatment, the cells were harvested, washed with PBS and incubated with culture medium (without FBS) containing JC-1 probes (2 mM) for 30 min at 37°C in the dark. Following the removal of the JC-1 probes, the cells were washed with PBS, harvested by trypsinization and resuspended in PBS. The amount of JC-1 retained by 10,000 cells per sample was measured at 530 nm (green fluorescence) and 590 nm (red fluorescence) with a flow cytometer and analyzed with CellQuest Alias software (BD Biosciences). CCCP-treated samples were used to perform standard compensation analysis. Data are presented as the ratio of red to green signals (590/530 nm).

### Statistical analysis

Each value is expressed as the mean ± standard deviation from at least three independent experiments. The differences in the means between groups were tested by one-way analysis of variance followed by the Student-Newman-Keuls test (comparisons among multiple groups). P<0.05 was considered to indicate a statistically significant difference.

## Results

### Effects of TMPZ on cell viability and apoptosis in SGC7901 cancer cells

**Figure 1.**
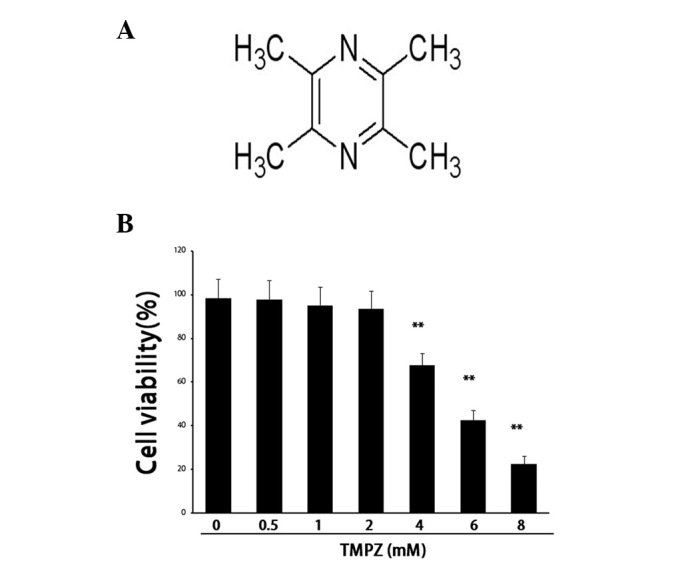
Effect of TMPZ on cell viability. (A) Structure of TMPZ. (B) SGC7901 gastric cancer cells were incubated with different doses of TMPZ for 24 h. Cell viability was determined by the MTT assay as described in Materials and methods. Data are expressed as the percentage of cell viability and represent the mean ± SD of three independent experiments. ^**^P<0.01 compared with control cells. TMPZ, tetramethylpyrazine; MTT, 3-(4,5-dimethylthiazol-2-yl)-2,5-diphenyl tetrazolium bromide.

**Figure 2.**
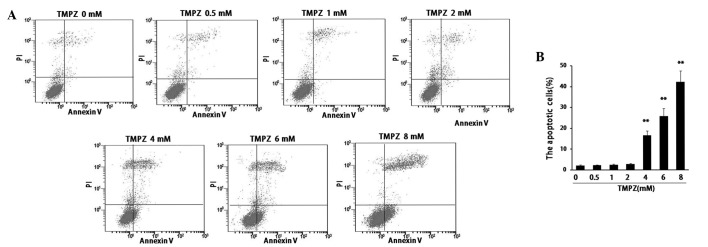
TMPZ induces apoptosis in SGC7901 gastric cancer cells. The cells were treated with different doses of TMPZ for 24 h and then stained with fluorescence-conjugated Annexin V and PI, and analyzed by flow cytometry. (A) Representative flow cytometric dot plots (x-axis, annexin V staining; y-axis, PI staining). (B) Quantitation of apoptotic cell population as shown in (A). The data are presented as the mean ± SD of three independent experiments. ^**^P<0.01 compared with the control cells. TMPZ, tetramethylpyrazine; PI, propidium iodide.

To investigate the antitumor effect of TMPZ on SGC7901 gastric cancer cells *in vitro*, cell viability and apoptosis were assessed. Following the administration of TMPZ (0–8 mM for 24 h) to SGC7901 cells, a contrasting effect of TMPZ towards tumorigenic cells was observed. Treatment with TMPZ at the higher doses of 4, 6 and 8 mM for 24 h resulted in reduced cell viabilities of 67.8±5.4, 42.6±4.5 and 22.5±3.5% in SGC7901 cells (P<0.01), respectively, whereas treatment with lower doses of TMPZ (0.5, 1 and 2 mM) demonstrated no effect on cell viability (Fig. 1). Furthermore, the analysis of apoptosis was concordant with the results observed in the cell viability experiments. The percentages of apoptotic cells were 16.7±2.1, 25.8±3.7 and 42.3±5.2% in SGC7901 cells treated with the respective doses of TMPZ (4, 6 and 8 mM) for 24 h (Fig. 2). These findings confirmed that a high dose of TMPZ confers significant cytotoxicity, which may be beneficial for anticancer treatment. Therefore, the higher doses of TMPZ (4, 6 and 8 mM) were selected for further experimentation.

### TMPZ initiates the accumulation of ROS in a dose-dependent manner

**Figure 3.**
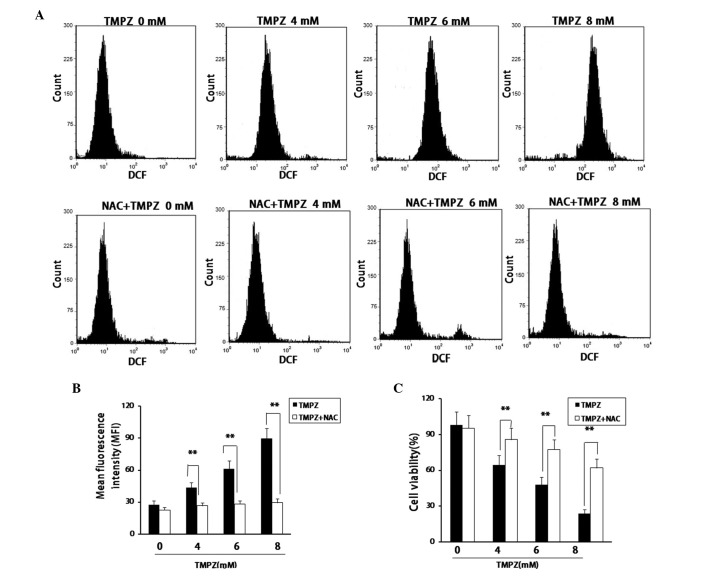
ROS mediates cell death induced by TMPZ. SGC7901 cells were treated with the indicated concentration of TMPZ in the absence or presence of 5 mM NAC for 24 h. (A) TMPZ induces dose-dependent ROS accumulation. SGC7901 cells were treated with 4, 6 or 8 mM TMPZ for 24 h. Cells were stained with DCFH-DA and analyzed by flow cytometry. (B) Quantitative analysis of the percentage of ROS production determined by FACS analysis. Data are expressed as the mean fluorescence intensity and represent the mean ± SD of three independent experiments. ^**^P<0.01. (C) ROS mediate the reduction of cell viability induced by TMPZ. Cell viability was analyzed by the MTT method. The values represent the mean ± SD of three independent experiments. ^**^P<0.01. ROS, reactive oxygen species; TMPZ, tetramethylpyrazine; NAC, N-acetylcysteine; DCFH-DA, 2’7’-dichlorodihydrofluorescein diacetate; MTT, 3-(4,5-dimethylthiazol-2-yl)-2,5-diphenyl tetrazolium bromide.

A number of natural compounds for the treatment of cancer have been demonstrated to cause increased levels of cellular ROS generation (9,10). To determine whether this mechanism is responsible for the antitumor effect of TMPZ, the generation of ROS was investigated. The results demonstrated that TMPZ treatment significantly elevated the ROS levels in a dose-dependent manner in SGC7901 cells. These data were further confirmed by incubating the cells with NAC, which is a scavenger of ROS (11). Treatment with NAC almost completely abrogated the release of ROS in SGC7901 cells and inhibited TMPZ-induced cytotoxicity (Fig. 3).

### TMPZ-induced activation of AMPK is mediated by ROS

**Figure 4.**
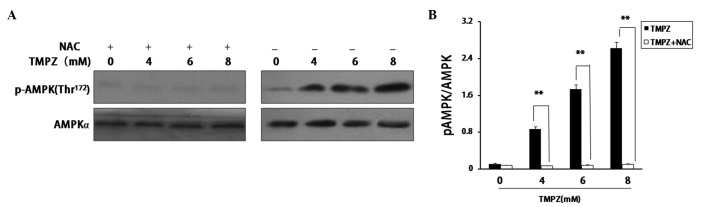
ROS mediates the activation of AMPK induced by TMPZ. SGC7901 cells were treated with the indicated concentrations of TMPZ in the absence or presence of 5 mM NAC for 24 h. The alterations in the levels of p-AMPK (Thr^172^) and AMPK were analyzed by western blot analysis. Representative images of at least three independent experiments are shown. (B) Levels of AMPK phosphorylation were quantified by densitometric analysis. The data are expressed as the ratio of p-AMPK to AMPK and represent the mean ± SD of three independent experiments. ^**^P<0.01. ROS, reactive oxygen species; AMPK, AMP-activated protein kinase; TMPZ, tetramethylpyrazine; NAC, N-acetylcysteine.

Numerous studies have implicated AMPK in cancer growth and metabolism (?). It has previously been suggested that stimulating the release of ROS may lead to the activation of AMPK (12); therefore, we investigated whether AMPK activation may result from TMPZ treatment. The results from this study revealed that high doses of TMPZ significantly upregulated the phosphorylation of AMPK in SGC7901 cells in a dose-dependent manner. To determine whether the activation of AMPK is involved in the accumulation of ROS, the cells were incubated with TMPZ in the presence of 5 mM NAC for 24 h. NAC almost completely inhibited TMPZ-induced AMPK phosphorylation, demonstrating that AMPK was activated by TMPZ-induced ROS accumulation (Fig. 4).

### TMPZ-induced activation of the mitochondrial apoptotic pathway is mediated by AMPK

**Figure 5.**
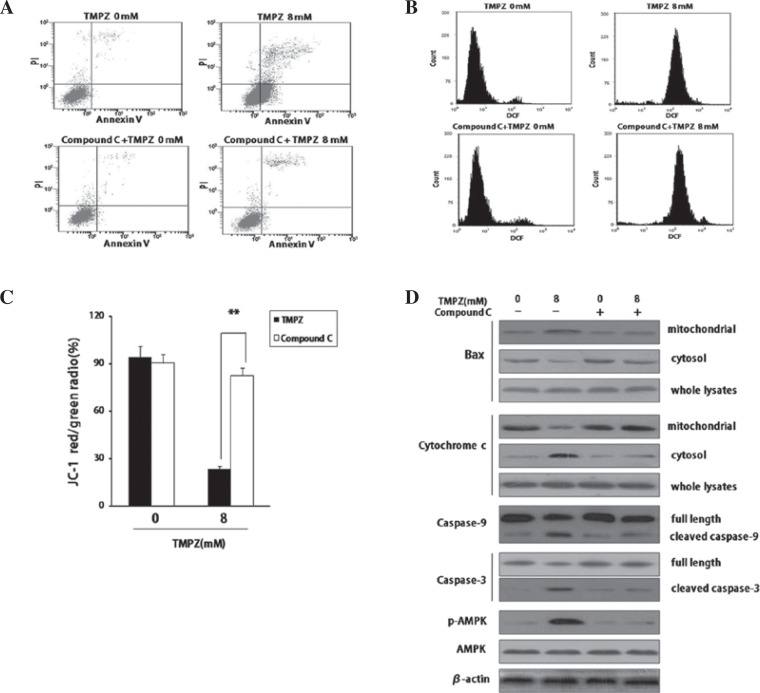
AMPK mediates TMPZ-induced apoptosis by initiating the mitochondrial apoptotic pathway. SGC7901 cells were treated with 8 mM TMPZ for 24 h with or without pretreatment with 10 *μ*M compound C. (A) Compound C mediates TMPZ-induced apoptosis. Following 24 h of treatment with TMPZ, cells were stained with PI and Annexin V, and analyzed by flow cytometry. Representative measurements from at least three independent experiments are shown. (B) Compound C does not suppress TMPZ-induced ROS accumulation. Cells were stained with DCFH-DA and analyzed by flow cytometry. Representative measurements are shown. The relative levels of ROS geometry fluorescence are shown as the mean fluorescence intensity. Each experiment was performed in triplicate. (C) AMPK mediates mitochondrial depolarization induced by TMPZ. Cells were stained with JC-1 and analyzed by flow cytometry. The ratio of JC-1 red/green fluorescence intensity was normalized by comparing the data to the control group and represents the loss of mitochondrial membrane potential. Each experiment was performed in triplicate and each value reported represents the mean ± SD. (D) AMPK mediates TMPZ-induced apoptosis by initiating the mitochondrial apoptotic signaling pathway. The mitochondrion and cytoplasm were separated, and proteins were extracted from each fraction. The alteration and subcellular localization of Bax and cytochrome *c* were analyzed by western blot analysis. In addition, caspase-9 and -3, pAMPK, AMPK and β-actin from whole cell lysates were analyzed by western blot analysis. ^**^P<0.01. AMPK, AMP-activated protein kinase; TMPZ, tetramethylpyrazine; PI, propidium iodide; ROS, reactive oxygen species; DCFH-DA, 2′7′-dichlorodihydrofluorescein diacetate.

To verify whether the AMPK signaling pathway was responsible for the antitumor effect of TMPZ that was observed in SGC7901 cells, compound C, an inhibitor of AMPK was used (13). Pretreatment with 10 *μ*M compound C for 15 min inhibited TMPZ-induced apoptosis (Fig. 5A). We also examined the mechanism by which AMPK mediated TMPZ-induced apoptosis. Compound C did not suppress TMPZ-induced ROS accumulation (Fig. 5B), indicating that compound C does not inhibit TMPZ-induced cell death by suppressing ROS accumulation. It is well-known that the majority of intrinsic death signals converge onto the translocation of Bax, a pro-apoptotic member of the Bcl-2 family, from the cytosol to the mitochondria (14). Our results demonstrated that TMPZ induced the translocation of Bax from the cytosol to the mitochondria and that this translocation was significantly inhibited by compound C. In addition, compound C significantly inhibited TMPZ-induced mPTP opening and depolarization of the mitochondria. The JC-1 red/green fluorescence intensity ratios were 23.4±1.5 and 82.7±4.7% in SGC7901 cells treated with 8 mM TMPZ alone and 8 mM TMPZ combined with compound C pretreatment, respectively (Fig. 5C). It is well-known that the majority of intrinsic death signals converge onto the translocation of Bax, a pro-apoptotic member of the Bcl-2 family, from the cytosol to the mitochondria (14). Our results demonstrated that TMPZ induced the translocation of Bax from the cytosol to the mitochondria and that this translocation was significantly inhibited by compound C. Furthermore, compound C significantly inhibited the TMPZ-induced release of cytochrome *c* and the activation of caspase-9 and -3 (Fig. 5D). Thus, ROS-induced AMPK activation is important in initiating the mitochondria-mediated apoptosis of SGC7901 cells treated with TMPZ.

## Discussion

The induction of apoptosis in malignant cells is an important mechanism of action for anticancer drugs (15,16). TMPZ, one of the active components of the Chinese herbal medicine Chuanxiong, has been traditionally used in the treatment of neurovascular and cardiovascular diseases. Recently, an increasing number of studies have indicated that TMPZ possesses anticancer properties (17,18). However, the function and mechanism of TMPZ in gastric cancer have yet to be elucidated. In the present study, we demonstrated that TMPZ induces cytotoxicity by causing the accumulation of ROS. The accumulation of ROS led to the activation of AMPK, which then induced apoptosis. The effects of TMPZ on the accumulation of ROS and apoptosis were observed in SGC7901 cells. Therefore, we conclude that ROS represent a novel intermediate in mediating TMPZ-induced cytotoxicity.

As a metabolic-sensing protein kinase, AMPK is important as an energy sensor, mainly in ATP-deprived conditions (19). It was previously reported that AMPK is phosphorylated and activated by LKB1 (20). However, we observed that AMPK was activated by TMPZ-induced ROS accumulation. Several studies have indicated that AMPK activation reprograms metabolism and also enforces a metabolic checkpoint in the cell cycle, which suggests that AMPK-activating drugs may be useful as cancer therapeutics (7,21). Recently, metformin has been shown to inhibit the growth of a wide variety of tumor cells in culture in an AMPK-dependent manner, and the activation of AMPK by aminoimidazole carboxamide ribonucleotide has been observed to suppress the growth of tumors (22,23). The results of our study demonstrated that AMPK is activated following treatment with TMPZ in SGC7901 cells. Furthermore, we also demonstrated that TMPZ-induced activation of AMPK is dependent on the release of ROS, as co-treatment of SGC7901 cells with NAC, a scavenger of ROS, inhibited the TMPZ-induced activation of AMPK.

The exact mechanism by which TMPZ-induced AMPK activation results in apoptosis is unknown. The majority of intrinsic death signals converge onto the activation of the mitochondrial apoptotic pathway (24,25). The mitochondria-dependent pathway for apoptosis involves the release of cytochrome *c* from the mitochondrion into the cytosol, a process that is controlled by Bcl-2 family members (26). Genetic and biochemical studies indicate that Bax functions as a major component of the intrinsic apoptotic pathway in mitochondria (27). Bax is an effective, multidomain, pro-apoptotic protein that resides in the cytoplasm as an inactive monomer in healthy cells. Upon treatment with apoptotic stimuli, Bax undergoes conformational activation leading to its translocation into the mitochondrion. Several studies have previously implicated the conformational activation of Bax as an early event that contributes to the release of cytochrome *c* from mitochondria and subsequent caspase cascade activation in the mitochondria-dependent apoptotic signaling pathway (28,29). In the present study, TMPZ induced the translocation of Bax from the cytosol into mitochondria. To investigate whether AMPK activation was accountable for the translocation of Bax in SGC7901 cells, compound C was utilized. The results confirmed that AMPK activation induced by TMPZ treatment was responsible for the translocation of Bax. In addition, AMPK activation induced by TMPZ treatment resulted in a disruption of the ΔΨm and mitochondrial swelling. This event was accompanied with the release of cytochrome *c* and subsequently induced the activation of the caspase cascade. This role of AMPK was further supported by data indicating that compound C significantly inhibited the loss of ΔΨm and the subsequent mitochondrial apoptotic pathway.

To the best of our knowledge, our results demonstrate for the first time that TMPZ induces apoptosis in a gastric cancer cell line. Additionally, our data provide the first evidence that TMPZ-induced apoptosis occurs via the accumulation of ROS. Accumulation of ROS levels leads to AMPK activation, which is important in apoptosis in gastric cancer cells through a signaling cascade that includes the translocation of Bax from the cytosol into mitochondria, resulting in mitochondrial dysfunction followed by cytochrome *c* release. The release of cytochrome *c* stimulates the activation of the caspase cascade. Therefore, these results contribute to the understanding of the cell apoptotic pathways induced by TMPZ, and to the development of TMPZ as a potential treatment for human gastric cancer.
